# Effect of MTA versus CEM apical plugs on fracture resistance of endodontically treated simulated immature teeth restored with cast metal posts: an in-vitro study

**DOI:** 10.1186/s12903-021-01641-w

**Published:** 2021-05-28

**Authors:** Ensieh Grayli, Abbas Dashtban, Leyla Shadan, Naser Behnampour, Elham Afshari

**Affiliations:** 1 Department of Edndodontics, Gorgan School of Dentistry, Golestan University of Medical Sciences, Gorgan, Golestan Iran; 2 Gorgan School of Dentistry, Golestan University of Medical Sciences, Gorgan, Golestan Iran; 3 Department of Prosthodontics, Gorgan School of Dentistry, Golestan University of Medical Sciences, Gorgan, Golestan Iran; 4 Department of Biostatics and Epidemiology, Health Management and Social Development Research Center, Golestan University of Medical Sciences, Gorgan, Golestan Iran; 5 Department of Pediatric Dentistry, Gorgan School of Dentistry, Golestan University of Medical Sciences, Gorgan, Golestan Iran

**Keywords:** Fracture resistance, Cast metal, Endodontic post, Bioceramics, MTA, CEM

## Abstract

**Background:**

Endodontically treated immature teeth which are restored with cast metal posts are of the most susceptible teeth to fracture. An apical plug is usually used as root end filling in order to seal the wide apical foramen. The current study was performed to evaluate the effect of different apical plug materials (MTA and Calcium enriched mixture cement) at varied thicknesses on fracture resistance of teeth restored with cast metal posts.

**Methods:**

A total of 40 extracted intact single-rooted human mandibular premolars (removed for orthodontic reasons) were used in the study. The coronal part of each tooth was removed and root canal preparation was performed. A size 4 Gates Glidden drill was used to enlarge the canal and was passed through the apical foramen in order to simulate an immature apex. Samples were randomly divided into 5 groups (n = 8) according to apical plug (control group: No plug, group MTA5: 5 mm MTA plug, group CEM5: 5 mm CEM plug, group MTA3: 3 mm MTA plug, group CEM3: 3 mm CEM plug). Post-space preparations were performed and cast metal post-and-cores were fabricated and cemented. Fracture resistance was assessed using a universal testing machine. Fracture thresholds were recorded and data were analyzed using One-way ANOVA and Dunnett’s T3 tests with significance level at *P* value < 0.05.

**Results:**

The analysis showed a significant difference of fracture resistance between groups (*P* value < 0.05). The mean fracture resistance of samples in control group was significantly lower than MTA5 (*P* value = 0.003). There was no significant difference between other groups (*P* value > 0.05).

**Conclusions:**

Within the limits of this study, the evidence indicated that placement of a 5 mm MTA apical plug increased the fracture resistance in simulated immature teeth which are restored with cast metal posts, compared to control group (gutta-percha and sealer). While the results were not as promising for a 3 mm MTA apical plug or either 3 or 5 mm CEM apical plug.

## Background

Endodontic and prosthodontic treatment of immature teeth has always been challenging for practitioners. All the three factors of being endodontically treated (apexification), immaturity, and being restored with cast metal post, are among risk factors for root fracture [1–4]. Therefore, endodontically treated immature teeth which are restored with cast metal posts are of the most susceptible teeth to fracture.

Achieving an appropriate apical seal which is essential for prevention of microorganism’s ingress, is challenging in immature teeth, due to the wide apical foramen [5, 6]. Different materials have been advocated as apical plug materials, among which bioceramic-based materials such as Mineral trioxide aggregate (MTA), BioAggregate, Biodentine, Calcium enriched mixture cement (CEM) are of the most popular materials [7]. The use of bioceramic-based materials has shown to decrease the fracture susceptibility compared to calcium hydroxide paste which was traditionally used for endodontic treatment of immature teeth before introduction of bioceramic-based materials [4, 8, 9].

If an endodontically treated tooth is severely damaged, restoration of the tooth structure usually requires a post-and-core system. Currently, there are several post or post and-core systems available using different materials and techniques such as metal cast posts, metal prefabricated posts, carbon fiber posts, glass fiber posts, and zirconia posts [10, 11].

Despite better esthetic results of recently developed posts such as glass fiber posts, and zirconia posts [10, 12], cast metal post-and-core systems have been a successful treatment option for the restoration of severely damaged endodontically treated teeth -especially posterior teeth [13, 14].

The current study was performed to evaluate the effect of different apical plug materials (MTA and CEM) at varied thicknesses on fracture resistance of teeth restored with cast metal posts and to the best of our knowledge, this is the first study to do so. The null was that there was no significant difference of fracture resistance between and within experimental and control groups.

## Methods

A total of 40 extracted intact single-rooted human mandibular premolars (removed for orthodontic reasons in dental clinic of Gorgan School of Dentistry and three private offices) were selected based on the inclusion criteria. Since it was the first study of its kind, sample size calculation was not applicable. Preparation of the samples was performed by a dental student (AD), guided by supervising endodontics and prosthodontics faculties (EG and LSh).

The teeth were disinfected with 5.25% NaOCl and stored in isotonic saline solution. Visual and stereomicroscope at 10× magnification (SMP-200, HP, USA) assessments were performed and teeth with a single straight root of similar length and size with no sign of crack, fracture, or carious lesion were selected.

**Fig. 1:**
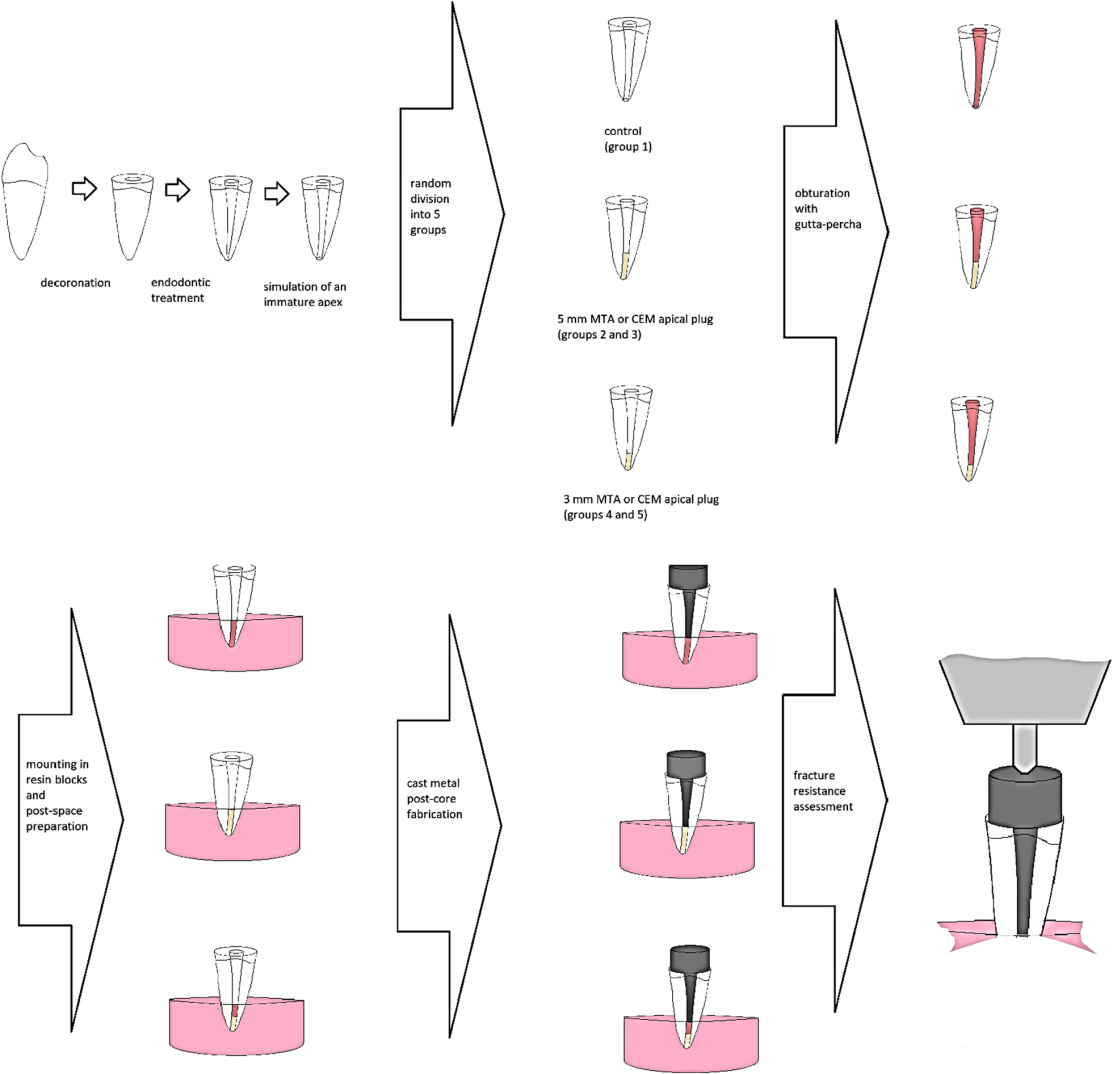
Samples preparation steps

A scheme of the samples preparation steps is shown in Fig. 1. The coronal part of each tooth was removed in a way that a 16 mm root was remained. Root canal preparation was performed using rotary NiTi Protaper instruments (Dentsply Maillefer, Ballaigues, Switzerland) with shaping and finishing files (Sx, S1, S2, F1 and F2). Canals were irrigated with 2 ml isotonic saline between preparation steps. A size 4 Gates Glidden drill was used to enlarge the canal and was passed through the apical foramen in order to simulate an immature apex. Samples were randomly divided into 5 groups using the simple randomisation method (a control group and 4 experimental groups) according to apical plug materials [mineral trioxide aggregate (MTA Angelus, Soluçoes Odontologicas, Londrina, Brazil), and calcium enriched mixture (CEM, BioniqueDent, Tehran, Iran)].

1. *Control group* No plug.
2. *Group MTA5* MTA orthograde 5 mm plug.
3. *Group CEM5* CEM orthograde 5 mm plug.
4. *Group MTA3* MTA orthograde 3 mm plug.
5. *Group CEM3* CEM orthograde 3 mm plug.

The materials were prepared according to the manufacturer's instructions (MTA was mixed at 1 spoon of MTA powder and 1 drop of sterile water and CEM was mixed at 3 portions of CEM powder and 1 drop of CEM liquid). The plugs placed in an orthograde manner, using MTA carrier (Dentsply Maillefer, Switzerland). In experimental groups, apical plug materials (MTA or CEM) were condensed using back end of thick paper points. In case of overfilling, the excess material was removed by a sterile blade. Correct placement was confirmed with preapical radiographs (Kodak, Carestream Health, USA). A wet paper point was placed in the root canal and samples were stored in wet gauze for 24 h. All root canals were obturated using gutta-percha (Meta Biomed Co. Ltd, Korea) and AH26 sealer (Dentsply, DeTrey, Germany) using cold lateral condensation technique with accessory gutta-percha cones. The samples were then sealed by temporary cement (Cavit, ESPE, Seefeld, Germany) and were incubated for 1 week at 37 °C.

The apical 5 mm of the samples were mounted in self-curing resin blocks, using a plastic ring mold (20 mm diameter and 10 mm thickness). Samples were vertically stabilized on surveyor, using plastic cylinders filled with acrylic resin. Post-space preparations were performed using preparation drills (D.T. Light-Post Universal Drill Size #0.5 for filling removal and D.T. Light-Post Finishing Drill Size #1 for post-space shaping). A minimum of 5 mm of apical seal was retained in all groups which means remaining only MTA/CEM in MTA5 and CEM5 groups, a combination of MTA/CEM and gutta-percha in MTA3 and CEM3 groups, and gutta-percha in the control group.

Resin patterns of posts were fabricated using acrylic resin (Duralay, Reliance Dental Mfg. Co., worth, Illinois) and direct technique. A 4-mm-height resin pattern for core was fabricated for all samples, using a standard mold made by cutting a plastic tube. Patterns were spurred, invested ((Deguvest lmpuls, Degu Dent Co., Germany), and casted using Ni–Cr alloy (T-3 Ni–Cr, CMP Industries LLC, NY, USA).

The castings were cleaned, sandblasted, and adjusted into the root canal. The cast posts were visually evaluated for adaptation. The castings were cemented with resin luting cement (Panavia F2.0, Kuraray medical Inc., OsaKa, Japan), using finger pressure. Samples were stored in saline solution at 37 °C for 3 days.

Fracture resistance was assessed using a universal testing machine (Zwik/Roell 020, Ulm, Germany). A static load was applied to each sample with a 90° angle to the occlusal surface of the metal core at a crosshead speed of 0.05 mm/min until the fracture occurred. Fracture thresholds were recorded and data were analyzed using SPSS 16.0 with significance level at *P* value < 0.05.

## Results

The mean, standard deviation, minimum, and maximum fracture resistance for each group is presented in Table 1. The values were normally distributed across all groups (Kolgomorov–Smirnov test, *P* > 0.05), so one-way ANOVA was used to identify the significant difference among the groups. Assumption of homogeneity of variance was rejected (Leven’s test, *P* < 0.05) and Dunnett’s T3 test was used for multiple comparisons.

**Table 1:** Mean and standard deviation of fracture resistance of values in 5 groups

Groups	N	Mean	Std. deviation	Minimum	Maximum
Control	8	1582.638	460.6348	1183.3	2256.3
MTA5	8	2916.950	637.5956	1925.2	3740.1
CEM5	8	2200.775	367.2977	1630.9	2906.2
MTA3	8	2105.438	228.4752	1802.6	2405.3
CEM3	8	2259.363	542.7525	1440.8	2900.1
Total	40	2213.033	619.2837	1183.3	3740.1

**Fig. 2:**
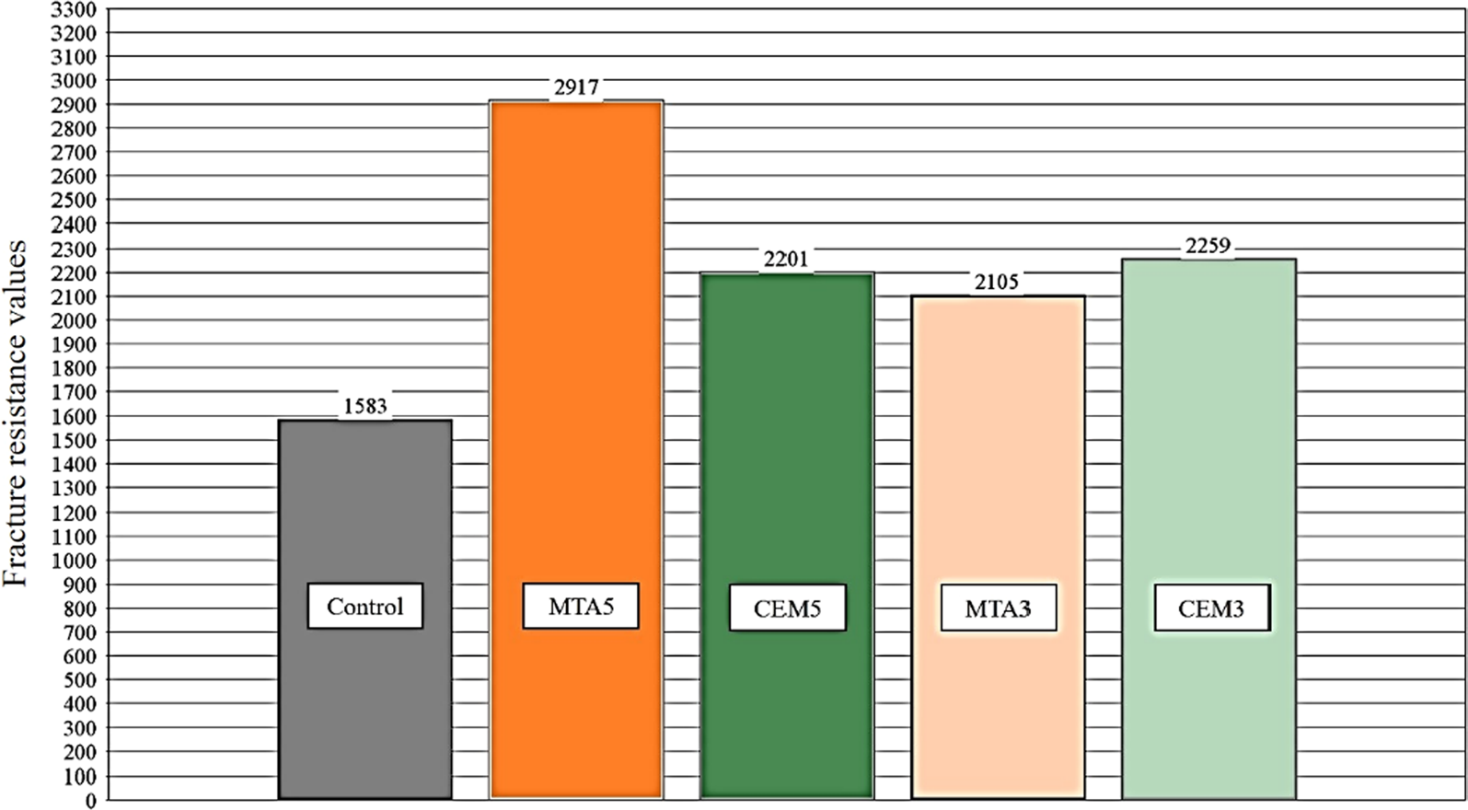
Comparison of fracture resistance values among groups

Using One-way ANOVA it was determined that there was a significant difference of fracture resistance between groups (*P* value < 0.05). The Dunnett’s T3 tests showed that the mean fracture resistance of samples in control group was significantly lower than MTA5 (*P* value = 0.003), and there was no significant difference between other groups (*P* value > 0.05) (Table 2). Figure 2 shows the comparison of fracture resistance values among groups.

**Table 2:** Intergroup comparison of fracture resistance (Dunnett test)

(I) group	(J) group	Mean difference (I–J)	Std. error	Sig	95% confidence interval	
					Lower bound	Upper bound
Control	MTA3	− 522.8000	181.7915	.125	− 1149.867	104.267
	CEM3	− 676.7250	251.6855	.145	− 1500.674	147.224
	MTA5	− 1334.3125*	278.0990	.003	− 2254.889	− 413.736
	CEM5	− 618.1375	208.2943	.089	− 1302.462	66.187
MTA5	MTA3	811.5125	239.4601	.066	− 44.383	1667.408
	CEM3	657.5875	296.0381	.313	− 311.419	1626.594
	control	1334.3125*	278.0990	.003	413.736	2254.889
	CEM5	716.1750	260.1528	.144	− 165.501	1597.851
CEM5	MTA3	95.3375	152.9332	.999	− 418.494	609.169
	CEM3	− 58.5875	231.7024	1.000	− 830.257	713.082
	control	618.1375	208.2943	.089	− 66.187	1302.462
	MTA5	− 716.1750	260.1528	.144	− 1597.851	165.501
MTA3	CEM3	− 153.9250	208.2010	.995	− 885.770	577.920
	control	522.8000	181.7915	.125	− 104.267	1149.867
	MTA5	− 811.5125	239.4601	.066	− 1667.408	44.383
	CEM5	− 95.3375	152.9332	.999	− 609.169	418.494
CEM3	MTA3	153.9250	208.2010	.995	− 577.920	885.770
	control	676.7250	251.6855	.145	− 147.224	1500.674
	MTA5	− 657.5875	296.0381	.313	− 1626.594	311.419
	CEM5	58.5875	231.7024	1.000	− 713.082	830.257

*The mean difference is significant at the 0.05 level

## Discussion

While endodontic treatment by itself increases the risk of root fracture [1], immature endodontically treated teeth are at an even higher risk of root fracture because of their thin dentinal walls [4, 5]. The situation becomes more complicated, if placement of an endodontic post is the treatment plan.

Traditionally, the long‐term use of calcium hydroxide has been the treatment of choice for induction of apexification in non-vital immature teeth [15]. However, the introduction of MTA in 1993 [16] provided a successful alternative with advantages including establishment of an instant apical barrier, setting ability in wet environment, promising sealing ability, provision of a shorter treatment period, improving patient compliance, ease of handling, and possibly increased fracture resistance of immature teeth [17–21]. CEM which was introduced in 2006 [22], has shown features comparable to those of MTA such as fracture resistance [4] and sealing ability [23]. CEM has even shown some superior results such as higher antibacterial effect [24], significantly shorter setting time, easier handling, and no tooth discoloration [25].

Although recently developed posts such as zirconia posts, carbon posts and glass fiber posts have offered better esthetic results than cast metal posts [5, 26], the superiority of success rate is controversial. Some studies have reported reduced chair-side and laboratory time of the new post systems [10, 12, 26], while others reported the advantages of cast metal posts such as higher retention [14] or superiority of results in special circumstances such as when multiple teeth need post systems or in case of tooth mal-alignment, and in small teeth with minimal dental tissue [27].

Overall, cast metal posts are still one of the most used systems, especially for the posterior teeth [28, 29]. Unfortunately, caries of young permanent teeth is still highly prevalent among children and adolescents in many countries [30–32]; and excessive tooth destruction due to dental caries is still a major reason tooth loss in these age groups [33, 34]. In case of severe tooth destruction, cast metal posts are still regarded as the gold standard for restoration [35], but previous studies concerning fracture resistance of endodotically treated immature teeth only focused on fiber post restorations [4, 36, 37]. Fiber posts have elastic modulus similar to that of the dentin [38], hence the results cannot be generalized to other post systems including cast metal posts.

In the present study, the effect of type and thickness of apical plug materials (MTA vs CEM/ 3 mm vs 5 mm) on fracture resistance of endodontically treated immature teeth restored with cast metal posts was assessed. Several studies have shown that a full canal obturation or an apical plugging by bio-ceramics, increases the fracture resistance of either mature or simulated immature teeth compared to the roots which were instrumented but were not filled, or were filled only with gutta-percha and sealer [8, 39, 40]. Full canal obturation by bio-ceramics is not indicated when placement of an endodontic post is the treatment plan, because further removal of the material for post-space preparation might not be easy [41], and also the material is more expensive than gutta-percha and sealer. Therefore, in such cases, MTA or CEM are used as apical plugs and the rest of the root canal is filled with gutta-percha and sealer [42, 43].

The results of the current study showed that the fracture resistance of samples in all experimental groups were higher than the control group (gutta-percha and sealer). However, the superiority was not statistically significant except for teeth filled with a 5 mm MTA apical plug.

While this is the first study to compare the effect of MTA and CEM apical plugs on fracture resistance of teeth restored with cast metal posts, effect of the two materials on fracture resistance of teeth has been compared in other circumstances and controversial results were reported. Evren et al. [4] compared the fracture resistance of simulated immature human teeth using 4 mm apical plugs (MTA, CEM, and Biodentine), with fiber post and composite resin restoration. They reported no statistically significant difference of fracture resistance between the experimental groups, which is in accordance with the results from the current study. Evren et al. also reported that fracture resistance values for all experimental groups were significantly higher than the control group, while in the current study, the difference was only significant for MTA5 group. The difference between the results regarding the comparison of the experimental and control groups may be explained by methodological differences between studies (i.e., preparations of the control groups, thickness of the plugs, type of post systems, and restorative materials).

Sarraf et al. [44] compared the fracture resistance of immature bovine teeth completely filled with MTA, CEM, and Biodentine with no post-space preparation and placement. They reported that MTA and Biodentine showed superior results over CEM. These results seem to be inconsistent with the results of the current study which showed similar fracture resistance values for both CEM and MTA. However, these differences could also be explained by methodological differences between studies, particularly in using full canal obturation or apical plug. Sarraf et al. also reported that the fracture resistance was not different for CEM, gutta-percha and sealer, and control (dried cotton wool filling) groups. The difference between results regarding the comparison of the experimental and control groups in the two studies can also be explained by different preparations of control groups and the thickness of obturations.

Milani et al. [8] also compared the fracture resistance of simulated immature human incisors filled with MTA, CEM, and MTA plus composite resin with negative control (untreated teeth) and positive control (unfilled teeth) groups. The results of this study showed no significant differences among three experimental groups, which appears to be in consistent with the present study. Milani et al. also reported no significant difference among MTA plus Composite and CEM groups with positive and negative control groups. While MTA group had significantly higher strength values than positive control. Difference in results regarding the comparison of the experimental and control groups in the two studies is observed; which can be related to different preparations of the control groups and use of post systems.

The current study also showed no statistically significant difference of fracture resistance regarding the thickness of the apical plugs (3 mm or 5 mm). Although several studies have been performed to compare the effect of using different thicknesses of apical plugs on root- end sealing ability [45–48], the studies assessing the effect of thickness on the apical of mechanical properties are rare. Madani et al. [48] compared the fracture resistance of simulated immature teeth, filled with 3 and 5 mm apical plugs of MTA and CEM with a control group (5 mm gutta-percha). Teeth were restored with glass fiber post and composite resin. As consistent with the current study, Madani et al. reported no statistically significant difference of fracture resistance between the experimental groups. However, unlike the present study, no significant differences from the control group was found; which can be attributed to the fact that the studies used different post systems and restorations.

The effect of thickness on surface micro-hardness of MTA and CEM has also been evaluated in several studies. A study performed by Tabrizizadeh et al. [49] showed no statistical difference of surface micro-hardness between 4 and 8 mm MTA and CEM plugs. Login et al. [50] also reported no statistic difference of surface micro-hardness between 4 and 6 mm MTA plugs, while 10 mm plugs were significantly harder that 4 and 6 mm plugs. Although the results of both mentioned studies appear to be in consistent with the present study, testing variable mechanical properties (i.e., surface micro-hardness and fracture resistance), prevents accurate comparison.

Root-end sealing ability, in addition to mechanical resistance and hardness, should be noticed while comparing different apical plug materials and thicknesses. Adel et al. [45] who compared the root-end sealing ability of different thicknesses of MTA and CEM, reported a significantly higher sealing ability of 5 mm apical plugs compared to 3 mm apical plugs of both materials. Valois et al. [46] and Gosh et al. [47], also reported a higher root-end sealing ability of 4 mm MTA plugs compared to lower thicknesses. Thus, decisions for clinical use of apical plugs should not be made only on the basis of mechanical properties. And comprehensive in-vitro and in-vivo studies of various factors influencing the clinical outcome of endodontics and prosthodontic treatment of immature teeth is required.

Overall, the results of most studies on fracture resistance are not comparable to each other, because of the great methodological variations regarding sample type (e.g. bovine/human teeth, premolars/incisors), sample preparations (e.g. immaturity simulation, root canal preparation and obturation techniques, post-space preparation, post systems), obturations (e.g. full canal/ apical plug, material thickness), coronal restorations (e.g. composite resins, metal cores or crowns), testing machine (fatigue/static load, speed, angle) and several other factors. Hence, there is a need to standardize the methods, in order to perform fair comparisons and interpretations.

In the current study, no crowns were placed on the cores, as in some other studies [51, 52], in order to avoid the confounding effect of assemblage of several adhesively bonded parts. Although use of crowns could be more similar to the clinical situation, and the researchers could limit the confounding effect by considering the mode of failure. Evaluation of the mode of failure of the specimens could also bring more information about the mechanisms and reasons of failures. Therefore, not assessing the failure mode is noted as a limitation of the current study and it can be suggested to the future researchers to conduct the assessment. Findings of the current study is based on a relatively small sample size which can also be considered as a limitation. Other limitations of the study are that exact same post-space dimensions could not be achieved because of operator dependency of the preparations and slight anatomic differences. However, size of the apical opening could be measured which can be considered in future researches. And finally, the limitations of an in-vitro, static fracture resistance test are understood.

## Conclusions

Within the limits of this study, the evidence indicated that placement of a 5 mm MTA apical plug increased the fracture resistance in simulated immature teeth which are restored with cast metal posts, compared to control group (gutta-percha and sealer). While the results were not as promising for a 3 mm MTA apical plug or either 3 or 5 mm CEM apical plug.

## Data Availability

The datasets used during the current study are available from the corresponding author on reasonable request.
